# Comparing the risk of severe oral mucositis associated with methotrexate as graft-versus host-disease prophylaxis to other immunosuppressive prophylactic agents in hematopoietic cell transplantation: a systematic review and meta-analysis

**DOI:** 10.1007/s00520-024-08722-w

**Published:** 2024-07-17

**Authors:** Aisha A. H. Al-Jamaei, Joel B. Epstein, Jan G. A. M. de Visscher, Ricardo T. Spielberger, Ryotaro Nakamura, Judith E. Raber-Durlacher

**Affiliations:** 1 Department of Oral and Maxillofacial Surgery, Amsterdam UMC, Amsterdam, The Netherlands; 2 Department of Oral Medicine, Periodontics, Diagnostic, and Oral Radiology, Faculty of Dentistry, Sanaá University, Sanaá, Yemen; 3 Department of Oral Surgery and Oral Medicine, Al-Razi University, Sanaá, Yemen; 4 Cedars-Sinai Medical Center, Los Angeles, CA USA; 5 City of Hope Comprehensive Cancer Center, Duarte, CA USA; 6 Department of BMT, Southern California Permanente Medical Group, Kaiser Permanente, Los Angeles, CA USA; 7 Department of Hematology &, Center for Stem Cell Transplantation at City of Hope Comprehensive Cancer Center, Hematopoietic Cell Transplantation, Duarte, CA USA; 8 Department of Oral Medicine, Academic Centre for Dentistry Amsterdam, Amsterdam, The Netherlands

**Keywords:** Methotrexate, Oral mucositis, Graft versus host disease, Hematopoietic cell transplantation, HCT

## Abstract

**Purpose:**

This study examines the risk of severe oral mucositis (SOM) in graft-versus-host disease prophylaxis (GVHD) compared to other agents in hematopoietic cell transplantation patients.

**Methods:**

A comprehensive search of four databases, including PubMed, Embassy, Web of Science, and Scopus, was conducted to identify studies reporting frequency and severity of oral mucositis in association with GVHD prophylactic regimens. RevMan 5.4 was used to perform the meta-analysis. Risk of bias assessment was carried out using the Rob-2 tool for randomized clinical trials (RCTs) and ROBINS-I tool for observational studies.

**Results:**

Twenty-five papers, including 11 RCTs and 14 observational studies, met the inclusion criteria. The pooled results from eight RCTs showed a higher risk of SOM in patients receiving MTX or MTX-inclusive GVHD prophylaxis versus non-MTX alternatives (RR = 1.50, 95% CI [1.20, 1.87], *I*^2^ = 36%, *P* = 0.0003). Mycophenolate mofetil (MMF) and post-transplant cyclophosphamide (Pt-Cy) consistently showed lower risk of mucositis than MTX. Folinic acid (FA) rescue and mini-dosing of MTX were associated with reduced oral mucositis severity.

**Conclusion:**

Patients receiving MTX have a higher SOM risk compared to other approaches to prevent GVHD, which should be considered in patient care. When appropriate, MMF, FA, and a mini-dose of MTX may be an alternative that is associated with less SOM. This work also underlines the scarcity of RCTs on MTX interventions to provide the best evidence-based recommendations.

## Introduction

Allogeneic hematopoietic cell transplantation (allo-HCT) is a procedure used to treat patients with a variety of hematological disorders, involving transplantation of stem cells or bone marrow from matched healthy siblings, or alternative donors [1, 2]. This procedure has demonstrated a remarkably improved overall survival (OS) and disease-free survival (DFS) in patients with hematological malignancies [2–4]. The preparatory regimen for these patients may include intensive chemotherapy and chemo-radiotherapy in order to eradicate the malignant cells prior to infusion of the graft, which can lead to serious complications [2, 5, 6]. One of the most well-known and serious complication of allo-HCT is graft-versus-host disease (GVHD), which can result in significant morbidity and mortality [7, 8]. Due to this, various prophylactic measures have been suggested to be included in the transplantation protocols. Despite the effectiveness of these approaches in reducing the incidence and severity of GVHD while preserving the graft-versus-tumor effect, they often come with the downside of causing toxicity in HCT recipients [9, 10].

One of the traditional standards for GVHD prophylaxis is the combination of calcineurin inhibitors (CNI) and methotrexate (MTX) [11]. However, a common toxic effect of this regimen is damaging the oral mucosal barrier resulting in oral mucositis [12]. This effect has been suggested to relate to direct MTX toxicity, which has been shown to concentrate in epithelial cells and cause epithelial cell necrosis [13, 14]. A general pathophysiological model was suggested explaining the complexity of the development of oral mucositis, which involves a series of biological interactions between the epithelium and the submucosal connective tissue [15]. The model suggests that the initiation stage is characterized by toxicity caused by chemo-and/or radiotherapy. These treatment modalities induce basal and suprabasal cell death either through direct DNA damage or generation of free radicals of reactive oxygen species (ROS). This primary damage leads to the second stage, which promote upregulation of several genes associated with pro-inflammatory cytokines, that in turn targets connective tissue and basal epithelium. The third stage, known as signal amplification, involves production of proinflammatory cytokines, providing positive feedback, thereby enhancing cytokines production that mediates further tissue damage. The fourth stage is characterized by ulceration, during which the lesion is subjected to microbial colonization. The final stage is healing, involving epithelial proliferation and restoration of epithelial integrity [15].

Developing severe oral mucositis (SOM) in patients undergoing HCT is a major contributor to decreased quality of life and increased treatment-related mortality [16, 17]. SOM is associated with various issues, including pain, dysgeusia, difficulty with eating and speaking, inability to take medications, prolonged hospital stay, parenteral nutrition, and high risk of infection and sepsis [18, 19]. In HCT patients who are submitted to GVHD prophylaxis, it is well-documented that SOM is primarily influenced by the type and intensity of the conditioning regimen [20, 21]. However, it may be exacerbated by GVHD prophylactic agents, such as MTX.

The introduction of various combinations of different immunosuppressive drugs, such as mycophenolate mofetil (MMF), sirolimus, and corticosteroids, as GVHD prophylaxis has shown promising capacity in reducing the frequency and severity of oral mucositis. Previous systematic reviews focused on comparing the effect of MTX vs. MMF as prophylaxis for GVHD, including their impact on the development of SOM [22, 23]. These reviews showed that the risk ratio of severe mucositis was significantly higher in association with MTX than with MMF. To the best of our knowledge, despite MTX being considered as the gold standard for GVHD prophylaxis in practice, there is no study providing a comprehensive overview of MTX-related oral mucositis and comparing this adverse effect to the other GVHD prophylactic medications. Therefore, the primary objective of this systematic review was to evaluate the risk of SOM in association with MTX and to explore whether or not there is an alternative prophylaxis equivalent to MTX but with fewer occurrence of SOM.

## Materials and methods

### Protocol and registration

**Fig. 1:**
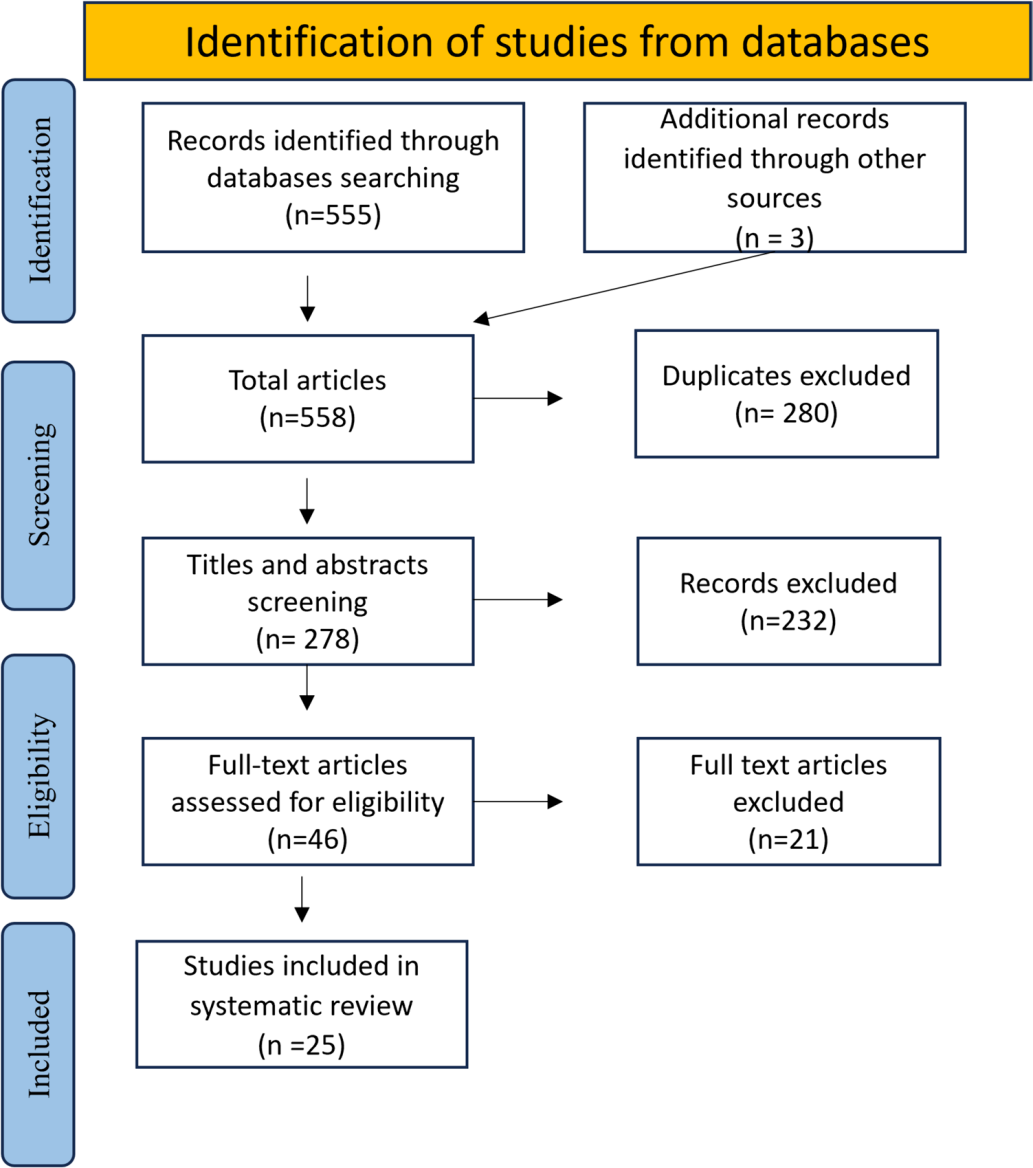
Flow chart depicting the selection process of the included studies. Reviews and preclinical studies as well as clinical studies that provided no outcome of interest were excluded

This review was designed and reported following the guidelines of the Preferred Reporting Items for Systematic Reviews and Meta-analysis (PRISMA) checklist [24] (Fig. 1). The protocol was also registered in PROSPERO bearing the registration number (CRD42023441298).

### Search strategy

A systematic and comprehensive search was conducted to explore PubMed, Embase, Web of Science, and Scopus without any restriction for the time of publication, using combination of following terms: “Oral mucositis,” oropharyngeal mucositis,” “oral stomatitis,” “Graft versus host disease,” “GVHD,” ”prophylaxis,’ “hematopoietic cell transplantation,” and “Methotrexate.” Results were also supplemented with manual search for the references list of relevant studies to identify additional relevant citations. The initial search was conducted in July 2023 and was updated in December 2023. Only sources in English were considered for inclusion.

### Study selection

The first and last authors worked independently to examine the retrieved titles and abstracts. Studies that were clearly irrelevant were immediately excluded. Full-text articles were assessed against prespecified eligibility criteria. Any disagreement was resolved by discussion or consultation with the other authors.

#### Eligibility criteria

PICO framework was used to determine the inclusion and exclusion criteria

The PICO guidelines were as follows:

- P (Population): Population receiving MTX during allo-HCT as prophylaxis for GVHD
- I (Intervention): MTX and alone or as part of the administered prophylactic regimen
- C (Comparison): Comparison group receiving different doses of MTX or submitted to prophylaxis other than MTX
- O (Outcome): Developing severe and frequency of oral mucositis/stomatitis

Studies were considered eligible if they met the following criteria:

1. Clinical trials or observational studies assessing the efficacy of MTX for the prevention of GVHD in populations that underwent allo-HCT
2. Studies that clearly evaluated the development of oral mucositis
3. Studies including an intervention group with MTX
4. Studies using a clearly described and validated measuring tool to assess the severity of mucositis, like the World Health Organization (WHO) scale or the Oral Mucositis Assessment Scale (OMAS)

Studies were excluded if:

1. The study did not include an intervention with MTX
2. Studies evaluated efficacy of MTX as GVHD prophylaxis but not evaluated the development of oral mucositis or its severity
3. Uncontrolled studies
4. Inappropriate sources like reviews, letters, editorial articles, study protocols, or case reports
5. Studies published in a language other than English

### Data extraction

The following information was extracted from the eligible full-text studies to a specially designed data extraction form and categorized as follows: author and year of publication, study design, sample size, interventional medication(s) (methotrexate and all its regimen comparators), conditioning regimen, oral mucositis (incidence, severity, assessment scale, and duration), and outcome with emphasis on the severity of mucositis.

Currently, there is not a universal single scale for precisely scoring severity of oral mucositis [25]. As such, the studies we included utilized different scales for diagnosis and grading SOM, including World Health Organization (WHO) [26], Oral Mucositis Assessment Scale (OMAS) [27], and the National Cancer Institute Common Terminology Criteria for Adverse Events (NCI-CTCAE) [28]. The severity of oral mucositis is not consistently represented across these scales because they use different parameters. For example, the WHO scale evaluates oral mucositis severity based on objective criteria, such as erythema or ulceration and the patient’s ability to eat. It grades the severity on a scale from 0 to 4. On the other hand, OMAS is an objective scale that quantitatively measures mucositis based on erythema and the dimensions of the ulcer at nine sites within the oral cavity. Erythema is graded on a scale ranging between 0 and 2, and ulceration is graded from 0 to 3. The total average score ranges from 0 to 5. The CTCAE also takes into account the anatomical sites of mucositis development. This scale is graded from 1 to 4. To perform a meta-analysis, we defined SOM in different ways, as either grade 2–4, grade 3 and grade 4, or a median of 0.5 to 2. This was based on the study’s definition of severity and the tool used for mucositis evaluation (details shown in Table 1).

**Table 1:** Characteristics and summary data of the included studies (*n* = 25)

Author, year, country		Type of study	No. sample	Age (median)	Conditioning regimens	Interventional drugs			Mucositis						Outcome
									Prevalence/or mean “assessment scale”		Severity		Duration		
						(MTX/c)	Comparator		(MTX/c)	Comparator	(MTX/c)	Comparator	(MTX/c)	Comparator	
MTX vs. cyclosporine (CsA)															
Storb, 1985, USA [29]		RCT	48	20–30 s	Not clearly mentioned	SD-MTX		CsA	88% Mild: 10 Mod: 6 Severe: 6 “Not clearly mentioned”	72% Mild: 11 Mod: 5 Severe: 3 *P* = 0.06	6 (27%)	3 (12%)	14 days	17 days	CsA is better
Torres, 1989, Spain [30]		RCT	57	30 s	MAC	SD-MTX followed by I.V FA		CsA	Mild: 3 (10%) Mod: 18 (58%) Severe: 10 (32%) “Not clearly mentioned”	Mild: 5 (19%) Mod: 13 (50%) Severe: 7 (27%)	10 (32%)	7 (27%)			No significant difference
MTX vs. sirolimus															
Cutler, (2005), USA [31]		Observational	54	40 s	MAC	SD-MXT/Tac		Sirolimus/Tac (ST)	Mild: 2 (8%) Mod: 10 (42%) Severe: 12 (50%) “OMAS”	Mild: 11 (37%) Mod: 17 (57%) Severe: 2 (7%)	12 (50%)	2 (7%) (*P* = 0.0002)	22 days	18 days (*P* = 0.07)	ST is better
Cutler, (2014), USA [32]		RCT	304	40 s	MAC	SD-MXT/ Tac		Sirolimus/Tac (ST)	0.47 (SD,50.40) “OMAS”	0.31 (SD,50.28) *P* < 0.001	0.96 (SD, 0.63)	0.70 (SD,0.51) *P* < 0.001	---		ST is better
Törlén, 2016, Finland [33]		RCT	209	50 s	MAC/RIC	SD-MTX/CAs		Sirolimus/Tac (ST)	--- “WHO”	7 (7%)	---	---	---		No significant difference
Garming Legert, 2021, Norway [34]		RCT	141	50 s	MAC/RIC	SD-MXT/ CsA		Sirolimus/Tac (ST)	G 1: 24 G 2–4: 44 “OMAS & WHO”**	G 1:30 G 2–4: 43	G 2-4 64% Peak 1.86 (SD 1.22)	G 2–4 58% 1.75 (SD 1.19; *P* = 0 .59)	OM peaked at day 10 and healed at 24 days in both groups		No difference
MTX + folinic acid (FA) vs. MTX															
Robien, (2006), USA [35]		Observational	311	40 s	MAC	SD-MTX/FA ≤ 400µg/day		SD-MTx FA >400µg/day	<400 µg 14.7 (11.7–18.5) =400 µg 18.0 (15.8–20.4) “OMI”	>400 µg 14.1 (10.9–18.2)	---	---	---		No difference
Gori, 2007, Italy [36]		RCT	122	30 s, 40 s	MAC	SD-MTX/FA no ice		SD-MTX/FA cryotherapy	2–4 grade: 48 (80%) “WHO”	50 (80%) *P* = 0.46	32 (53.3%)	29 (46.7%) *P* = 0.92	2–4 grade median (range) 11 days (*P* = 0.58)	Median 10 days	No benefit of cryotherapy
Sugita, 2012, Japan [37]		Observational	118	40 s	MAC/RIC	SD-MTX/ Systemic FA		SD-MTX/FA mouthwash (13.0%)	G 1: 9 (31.0%) G 2: 5 (17.2%) G 3: 3 (10.3%) G 4: 0 (0.0%) “ NCI-CTCAE”		3 (10.3%)	11 (18.3%)			SD-MTX/systemic FA is better
Kodama, 2015, Japan [38]		Observational	111	9 years	MAC/RIC	SD-MTX/FA LD: 4 times HD: 25 times		SD/MTX	---	---	--	---			No significant difference
Yeshurun, 2020, Israel [39]		RCT	52	40 s	MAC	SD-MTX/systemic FA		SD-MTX /Placebo	77.8% “WHO”	83.3 % *P* = 0.64	45.8	46.6% *P* = 0.98	4 (2-10) days	4 (3-6) days *P* = 0.43	No difference
Freyer, 2021, USA [40]		Observational	92	40 s	MAC	SD-MTX/systemic FA		SD-MTX without FA	Grade 2–4: 36 (78%) “NCI-CTCAE”	42 (91%); *P* = 0.15	19 (41%)	28 (61%)	6 days	10.5 days; *P* = 0.0004	SD-MTX/FA significantly reduced mucositis RR: 0.68 (0.45_1.03)
MTX vs. mycophenolate mofetil (MMF)															
Kiehl et al., 2002, Germany [41]		RCT	45	---	MAC	SD-MTX/CsA		MMF/CsA	8 (62%) “WHO”	7 (22%)	G3: 1 G4: 4	G3: 5	---	---	MMF is better *P* = 0.02
Bolwell et al., 2004, USA [42]		RCT	40	40 s	MAC	SD-MTX/CsA		MMF/CsA	---“OMAS”	---	Median 0.25 Sever 4 (21%)	Median 1.0 Sever 11 (65%)			MMF is better P = 0.008
Perkin, 2010, USA [43]		RCT	89	40 s	MAC/RIC	SD-MTX/Tac		MMF/Tac	Mild:19 (51%) Mod: 2 (6%) Sever: 0 (0.0%) “ NCI-CTCAE”	17 (52%) 0 (0.0%) 0 (0.0%)	25 (53%)	14 (33% *P* = 0.06	Median 1 day	0 day *P* = 0.05	MMF is better
Piñana et al., 2010, Spain [44]		Observational	145	50 s	RIC	SD-MTX/CsA		MMF/CsA	G 1: 27 (29%) G 2: 24 (26%) G 3: 21 (23%) G 4: 8 (9%) “WHO”	17 (33%) 7 (13%) 5 (10%) 0 (0.0%)	57%	23% (*P* < 0.001)			MMF/ CsA is better
Hamilton et al., 2015, USA [45]		Observational	241	40 s	MAC	MD-MTX/CsA		MMF/CsA (174)	76 “OMAS &WHO”**	---	36 (53%)	33 (19%) *P ****<*** 0.001 HR: 0.08 (0.03–0.22) *P ****<*** 0.001			MMF/CsA is better
Hamilton et al., 2023, USA [46]		RCT	96	40s	MAC	SD-MTX/Tac		MD-MTX/MMF/Tac	--- “WHO”	---	40 (82%)	27 (57%) *P* = 0.01	18 days	11 days *P* < 0.001	MD-MTX/MMF/Tac is better
SD-MTX vs. MD-MTX															
Przepiorka, 1996, USA [47]		Observational	30	36 years	MAC	Mini-dose MTX coupled with tacrolimus			G1: 7 (23%) G2: 23 (77%) “Not clearly mentioned”	---	---	---	---	---	Mini-dose is safe and effective for prevention of OM in matched unrelated donors
Przepiorka, 1999, USA [48]		Observational	30	42 years	MAC	Mini-dose MTX combined with tacrolimus			G1: 7 (23%) G2: 23 (77%) “Not clearly mentioned”	---	---	---	---	---	Mini-dose is safe and effective for prevention of OM in mismatched donors
Matsukawa, 2016, Japan [49]		Observational	208	40–50 s	MAC/RIC	SD-MTX/Tac		MD-MTX/Tac	46 (76.7%) “NCI-CTCAE”	11 (68.8 %) (*P ****=*** 0.53)	16 (26.7%)	2 (12.5 %) (*P ****=*** 0.33)	Median:13.0	Median: 4.5 days; (*P ****=*** 0.013)	SD-MTX/Tac prolonged mucositis OR, 0.094; 95 % CI, 0.0090– 0.98; *P ****=*** 0.048)
Lino, 2021, Japan [50]		Observational	35	50 s	MAC/RIC	MD-MTX/Tac			10/35 (29%) “NCI-CTCAE”		G 1: 8/10 G 2: 2/10		-----		Short-term MTX with Tac is safe and effective no grade 3 or 4 OM
Solodokin, 2020, USA [51]		Observational	48	50 s	MAC/RIC	MD-MTX/Tac		------	G 1,2, 11 (22.9) G 3/4 6 (12.5) “NCI-CTCAE”		6 (12.5 %)				Mini-dose is safe and adding FA might be beneficial
MTX vs. cyclophosphamide (Cy)															
Ying, 2011, USA [52]		Observational	157	---	MAC	MTX/CsA		Cy	--- “Not clearly mentioned”	---	14 (93.3%)	36 (25.3%)			Cy was significantly associated with lower rate of severe mucositis
Shouman, 2023, Egypt [53]		Observational	150	20s-30s	MAC	MTX/CsA		Cy	--- “Not clearly mentioned”	---	32 (42.7%)	19 (25.3%)	---	---	Cy was associated with lower rate of severe mucositis

*MTX/c*, methotrexate/its combination; *SD-MTX*, standard dose of methotrexate; *Tac*, tacrolimus; *RCT*, randomized clinical trial; *MAC*, myeloablative conditioning; *RIC*, reduced intensity conditioning; *FA*, folinic acid; *MD-MTX*, mini-dose of methotrexate; *OM*, oral mucositis; *OMAS*, Oral Mucositis Assessment Scale; *WHO*, World Health Organization; *OMI*, Oral Mucositis Index; *NCI-CTCAE*, National Cancer Institute Common Terminology Criteria for Adverse Events, **evaluation based on WHO scale., *G*, grade; *SD*, standard deviation; *RR*, relative risk; *OR*, odds ratio; *P*, *P* value

### Risk of *bias* assessment

Overall, risk of bias was evaluated independently by the first two reviewers, and any disagreement was solved by discussion.

Risk of bias assessment of the included randomized clinical trials was performed using the revised Cochrane risk of bias tool (ROB 2.0), which comprises six main domains: random sequence generation (selection bias), allocation concealment (selection bias), blinding of participants and personnel (performance bias), blinding of outcome assessment (detection bias), incomplete outcome data (attrition bias), and selective reporting (reporting bias). Based on these six domains, the studies were categorized as low risk, some concern, or high risk of bias. Indeed, the risk of bias generally corresponded to the worst risk of bias in any domain.

To evaluate risk of bias in non-randomized studies (observational studies), (ROBINS-I) assessment tool was used. This tool evaluates the quality of the study design in terms of seven specific bias domains: confounding, selection of participants, classification of interventions, deviations from intended interventions, missing data, measurement of outcomes, and selection of the reported result.

Figures of publication quality risk of bias assessment were created online through Robvis website (robvis (shinyapps.io).

### Data synthesis

Meta-analysis was performed with a random-effects model to estimate risk ratios (RR) and 95% confidence intervals (CI) incorporating heterogeneity within and between studies with RevMan 5.4 (Cochrane Review Group). Heterogeneity between studies was assessed by considering the *I*^2^ statistics; according to the Cochrane Handbook, an *I*^2^ estimate of 50–90% may interpret as evidence of substantial levels of heterogeneity. Publication bias and small study effects were assessed using funnel plot asymmetry testing. In case sufficient studies were available, subgroup analysis was conducted.

## Results

**Fig. 2:**
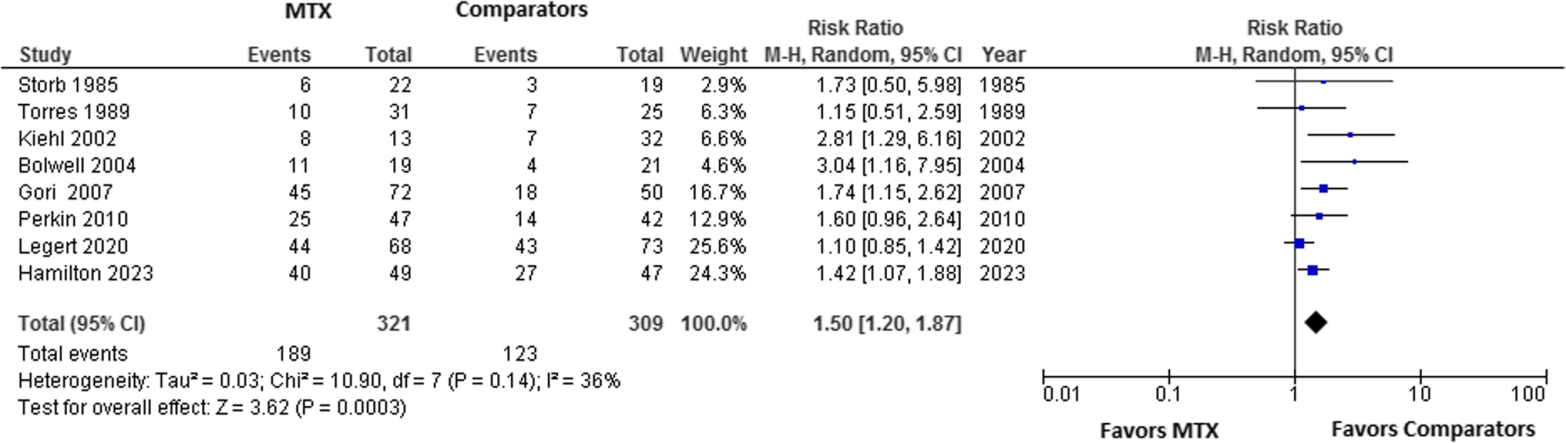
Forest plot of RCTs comparing risk of SOM in association with MTX vs. all comparators

As shown in Fig. 1, the electronic searches across the four databases initially retrieved a total of 555 articles, and 3 articles were identified manually from reference lists. After omitting duplicate records, the number of papers was reduced to 78. The full text of 46 articles was then assessed against prespecified eligibility criteria, resulting in 25 records that were considered for data extraction. Among these, there were 11 RCTs and 14 retrospective observational studies, with a collective sample of 2924 participants (Table 1). These studies were conducted across various geographic areas, including 12 studies in USA, four in Japan, one in Norway, one in Italy, one in Israel, one in Finland, one in Egypt, one in Germany, and three in Spain. The primary outcome focused upon evaluating the risk of development severe oral mucositis (SOM) in patients receiving MTX compared to the other prophylaxis medications. The meta-analysis of the pooled risk ratio (RR) estimates of eight RCTs revealed that the risk of SOM was significantly higher in MTX participants than in patients who received other prophylaxis, (RR = 1.50, 95% CI [1.20, 1.87], *I*^2^ = 36%, *P =* 0.0003) (Fig. 2).

**Fig. 3:**
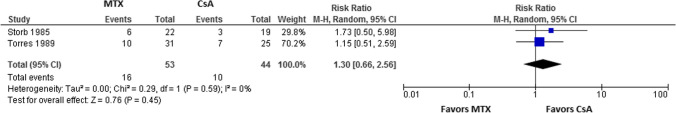
Forest plot of RCTs comparing risk of SOM in association with MTX vs. CsA

A subgroup analysis of two RCTs compared the development of severe mucositis in patients receiving MTX to those receiving cyclosporine as prophylaxis, and the result showed a non-significant higher risk of SOM in MTX group than in the cyclosporine group (RR = 1.30, 95% CI [0.66, 2.56], *I*^2^ = 0 %, *P* = 0.45) (Fig. 3).

**Fig. 4:**
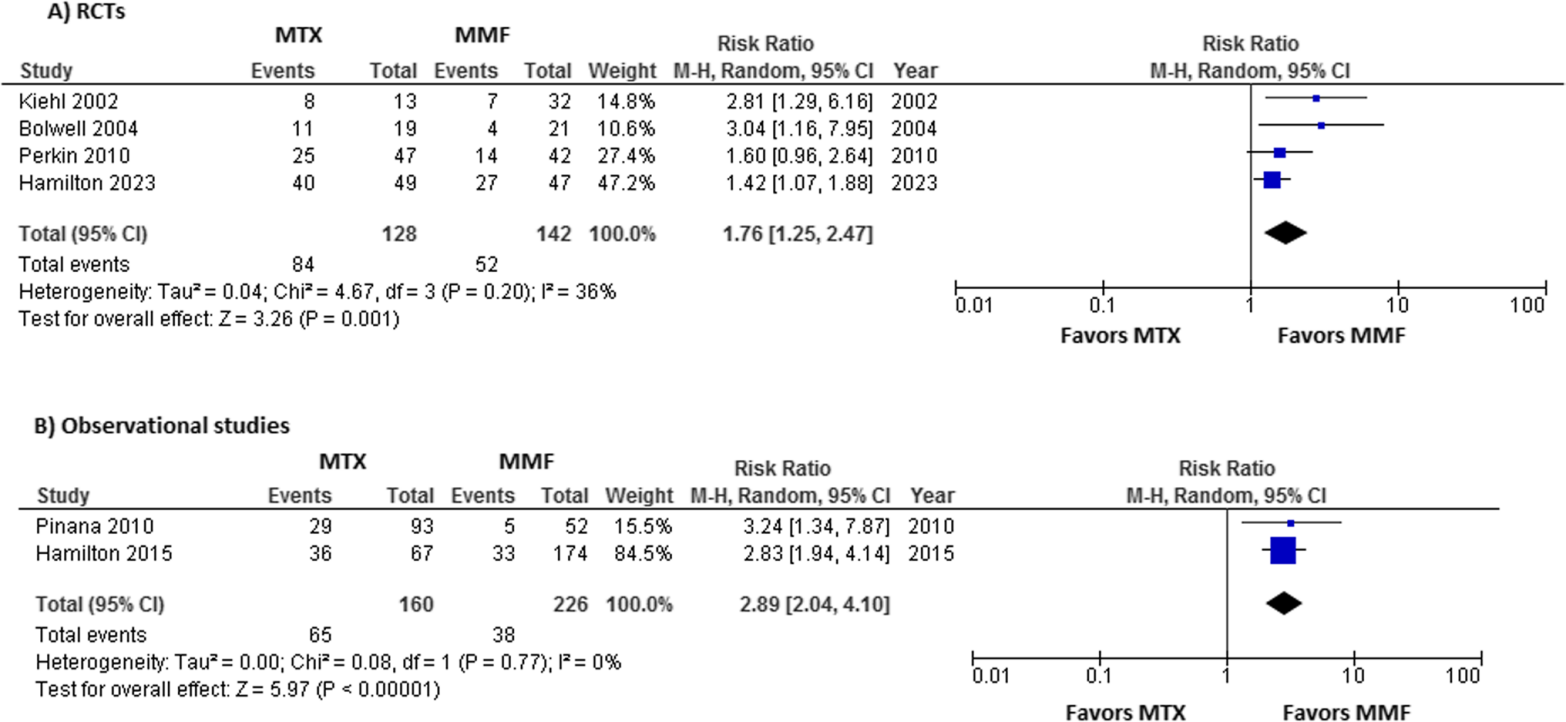
**A** Forest plot of RCTs comparing risk of SOM in association with MTX vs. MMF. **B** Forest plot of observational studies comparing risk of SOM in association with MTX vs. MMF

With respect to MTX vs MMF, we updated the analysis of a previous study and found a significant reduction of SOM in subjects treated with MMF (RR = 1.76, 95% CI [1.25, 2.47], *I*^2^ = 36%, *P* = 0.001) (Fig. 4a) [22]. We further conducted meta-analysis for two observational studies assessing incidence and severity of oral mucositis with MTX and MMF. In line with the results of RCT analysis, this provided evidence that MTX was more likely associated with higher incidence of SOM (RR = 2.89, 95% CI [2.04, 4.10], *I*^2^ = 0%, *P* < 0.00001) compared to MMF (Fig. 4b).

**Fig. 5:**
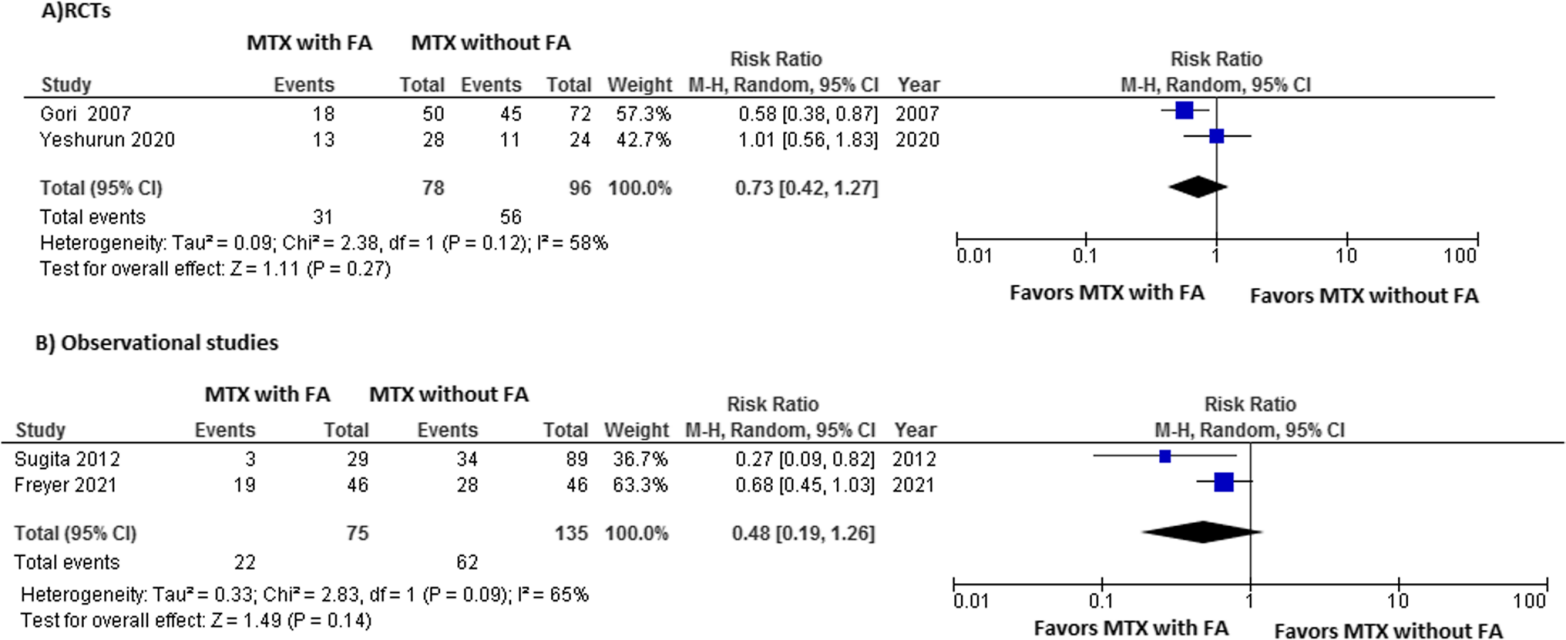
**A** Forest plot of RCTs comparing risk of SOM in association with MTX/FA vs. MTX without FA. **B** Forest plot of observational studies comparing risk of SOM in association with MTX/FA vs. MTX without FA

We also identified two observational studies and two RCTs evaluating folinic acid (FA) efficacy in reducing MTX oral toxicity. Meta-analysis of the RCTs showed a protective effect of FA in reducing the incidence of SOM between participants receiving MTX with FA and those receiving MTX without FA, although this was not significant (RR = 0.73, 95%CI [0.42, 1.27], *I*^2^ = 58%, *P*= 0.27) (Fig. 5a). Similarly, the meta-analysis of the two observational studies reported a non-significant beneficial effect of FA in combination with MTX against SOM (Fig. 5b). The application of cryotherapy had no added value in preventing oral mucositis in patients receiving MTX with FA as GVHD prophylaxis in allogeneic HCT [36].

Additionally, we identified four studies comparing the risk of mucositis between MTX and sirolimus and their combinations. Two of these studies showed no significant difference neither in incidence nor in severity and duration of oral mucositis between MTX and sirolimus groups [33, 34]. In contrast, the other two studies reported a significant reduction in severity and duration of oral mucositis in favor of sirolimus [12, 32].

Various studies evaluated the effectiveness of mini-dose MTX (MD-MTX) in reducing the incidence of severe mucositis while preserving good efficacy as GVHD prophylaxis (47-51). All these studies showed that MD-MTX was safe, effective, and can be used as an alternative for the standard dose of MTX (SD-MTX) in the prophylaxis protocol.

**Fig. 6:**
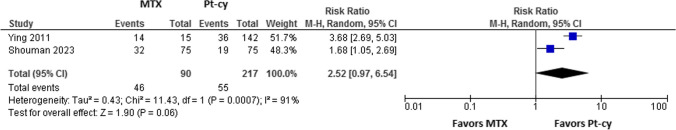
Forest plot of observational studies comparing risk of SOM in association with MTX vs. Pt-cy

Finally, two studies were identified that compared the prevalence of SOM in patients receiving MTX versus those receiving post-transplant cyclophosphamide (Pt-Cy) as GVHD prophylaxis. The meta-analysis suggested a remarkable increase in SOM among those treated with MTX, though non-significant (RR = 2.52, 95% Cl [0.97, 6.54], *I*^2^ = 91%, *P* = 0.06) (Fig. 6).

### Risk of *bias* analysis

**Fig. 7:**
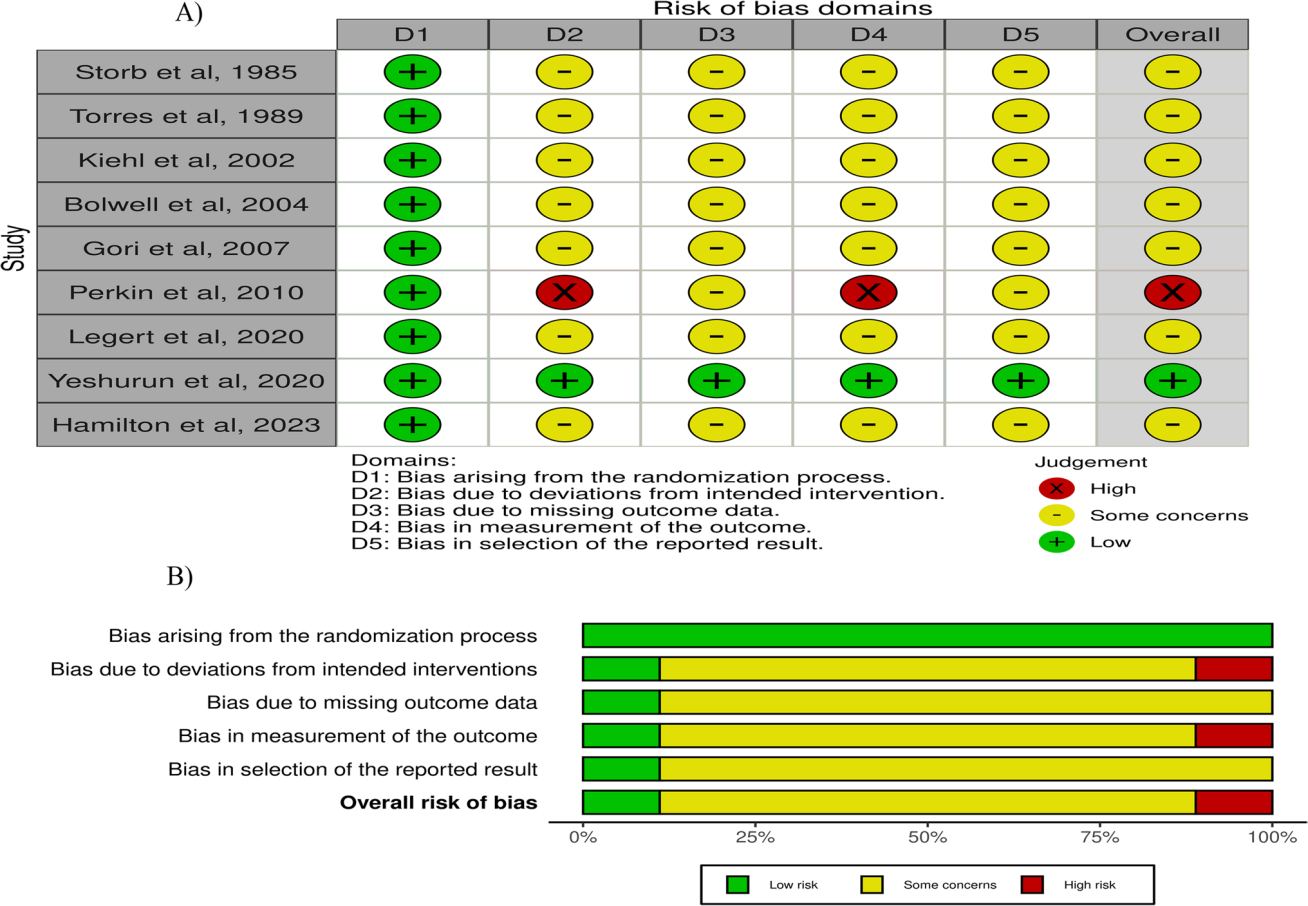
**A**) Risk of bias summary. **B**) Risk of bias graph

Risk of bias of RCTs was measured based on the Cochrane guidelines (RoB 2.0). The overall risk of bias was found to be high in one study [43], low in one study [39], and to have some concerns in the remaining seven studies [29, 30, 34, 36, 41, 42, 46] (Fig. 7). Proper randomization was performed in all RCTs and was assessed to have low risk of bias of this domain. Regarding domains 2, 3, 4, and 5, seven studies were rated as having some concerns [29, 30, 34, 36, 41, 42, 46], whereas one study was rated as having low risk [39]. One study was rated to have a high risk in domains 2 and 4, in particular [43].

Regarding observational studies, risk of bias was evaluated according to ROBINS-I tool (Table 2). Overall five studies were rate as having moderate risk of bias [37, 40, 44, 45, 53], and one study did not have enough information for risk assessment (52). In domain 1, four studies were rated as having a moderate bias risk [37, 44, 45, 52], while two studies were rated as low (40, 53). In domains 2, 4, and 5, five studies were rated low [37, 40, 44, 45, 53] and one [52] rated either moderate or no information. In domain 3, four studies were rated as low [37, 40, 44, 45] and two were rated moderate [52, 53]. None of the studies provided information on domains 6 and 7.

**Table 2:** Risk of bias in non-randomized studies according to the Risk of Bias in Non-Randomized Studies of Interventions (ROBINS-I) tool

Author	Domain 1	Domain 2	Domain 3	Domain 4	Domain 5	Domain 6	Domain 7	Overall
Pinana et al. [44]	Moderate	Low	Low	Low	Low	No information	No information	Moderate risk
Sugita et al. [37]	Moderate	Low	Low	Low	Low	No information	No information	Moderate risk
Hamilton et al. [45]	Moderate	Low	Low	Low	Low	No information	No information	Moderate risk
Freyer et al. [40]	Low	Low	Low	Low	Low	No information	No information	Moderate risk
*Ying et al. [52]	Moderate	Moderate	Moderate	No information	No information	No information	No information	No information
Shouman et al. [53]	Low	Low	Moderate	Low	Low	No information	No information	Moderate risk

^*^Available only as a conference abstract

## Discussion

The primary goal of GVHD prophylaxis protocols should be assessed while considering the toxicities associated with prophylaxis in order to provide guidance for the best patient care. The primary finding of our study indicates a significantly increased risk of developing SOM in patients receiving MTX and its combinations for GVHD prophylaxis compared to those receiving alternative prophylactic medications. We found that the incidence of SOM tended to be higher with administration of MTX in comparison to cyclosporine prophylaxis, although the increase was not statistically significant. Additionally, our analysis revealed a benefit of using FA in reducing oral toxicity associated with MTX use. Shifting to use MMF as alternative to MTX might be promising, as a significant and higher incidence of SOM was noticed in patients receiving MTX prophylaxis compared to those receiving MMF. Difference in incidence and severity of oral mucositis between MTX and sirolimus remains controversial. Further, our analysis consistently demonstrated that mini-dose MTX (MD-MTX) was safe and effective in reducing frequency and severity of oral mucositis compared to the standard dose MTX (SD-MTX) in the prophylaxis protocol. However, the indications for the use and the dose of MTX to reduce the sequelae of GVHD are diverse, and reducing the dose may not be possible. Finally, we found that patients who received Pt-Cy prophylactically demonstrated a reduced incidence of SOM compared to those who were administered MTX.

In light of enhanced understanding of pathophysiological mechanisms involved in GVHD, which is primarily mediated by T-lymphocytes, a multimodal prophylactic approach has focused on immunomodulatory agents that can suppress T-cells in transplant recipient [54, 55]. To date, the standard protocol for prevention of GVHD is combination of MTX with calcineurin inhibitors, such as cyclosporine or tacrolimus. Other immunosuppressive agents such as sirolimus and MMF are also commonly used in prevention of GVHD [56]. Our data and those of others revealed that SOM was a distressing side effect associated with all of these prophylactic agents. It also provided evidence that MTX and its combination induced SOM more frequently than other medications used. This indeed can be attributed to multifaceted action of MTX, which not only attenuates T-cells but also act as an antimetabolite and folate antagonist that directly inhibits proliferation of rapidly dividing cells, including oral epithelium [57]. Moreover, as stated above, MTX has been found to cause a direct and severe mucosal epithelial necrosis [13, 14]. This indeed can explain why oral mucositis is a common manifestation of MTX toxicity, in comparison to immunosuppressive drugs, which act on the immune system.

Following several promising pilot studies that demonstrated potential effectiveness of cyclosporine as a monotherapy in prevention of GVHD, two RCTs were conducted in the 1980s comparing the efficacy of cyclosporine and MTX [29, 30]. The results of both studies found no differences in the overall survival and prevention of acute GVHD, but variations were observed in other parameters. In terms of SOM, our pooled result of both studies showed a non-significant increase in the rate of SOM in the MTX group compared to the cyclosporine group. Nonetheless, it is important to note that one of these studies combined MTX with FA, which may have mitigated MTX toxicity [30]. Given the small number of trials and concern regarding risk of bias, these results might be unreliable and further well-designed studies are warranted.

It has been suggested that combination of FA with MTX can circumvent the dihydrofolate reductase, thereby restoring the folate function, and eventually reducing MTX toxicity, including SOM [58]. Our review supports this notion, as two RCTs revealed a protective effect of adding FA to MTX, which had led to much less events of mucositis [36, 39]. Intriguingly, a recent systematic review evaluated FA rescue after MTX administration in pediatric lymphoblastic leukemia patients and found a lower incidence of SOM when higher cumulative FA doses were administered early after the administration of MTX [59]. This underscores the importance of timing and dose to achieve a significant impact. Indeed, in our included studies, we found various protocols were followed regarding dose and timing which could explain the non-significant results in the observational studies. Additionally, it was reported that not only the systemic administration of FA rescue after MTX GVHD prophylaxis has a positive impact on regimen-related toxicity, including severity and duration of oral mucositis, but also a protective effect of a FA mouthwash was found [37]. This suggests a general beneficial effect of FA in reducing SOM. It is worth mentioning that no effect was found by using cryotherapy before infusion of MTX/FA [36]. This contrasts to a recent meta-analysis that revealed a significant effect of cryotherapy in preventing mucositis during chemotherapy for solid tumors and during conditioning with myeloablative chemotherapeutic agents such as melphalan in HCT setting [60, 61]. Further studies are needed to explore the effectiveness of cryotherapy with a MTX/FA protocol.

In line with previous work that found a significant reduction of SOM in patients submitted to MMF vs. those submitted to MTX, we observed a significant reduction in severity and duration of oral mucositis with using MMF as well. Our study not only updated the meta-analysis of Kharfan-Daban and coworkers [22] but also included two observational studies. The results consistently show superiority of MMF over MTX in reducing SOM, especially in matched related and unrelated donors. In fact, the association between mucositis and MTX can be easily explained by MTX mechanism of action on folate and consequently on DNA synthesis, which is critical in high turnover tissues like the oral mucosa, where there is a dose-related effect. Moreover, MMF is known to act as selective inhibitor of inosine monophosphate dehydrogenase which is a key enzyme in the critical pathway of T and B lymphocytes, indicating no direct link to oral mucosal damage, which is merely regulated by the innate immune system [62]. However, some case reports proposed that the association between mucositis and MMF could be the result of direct MMF-induced cytotoxicity [63]. Thus, monitoring the plasma concentration of MMF after infusion might help in reducing its cytotoxic effect; even though MMF has been found to be favorable with respect to SOM, this drug is not yet recommended to replace MTX, as it is not superior to it in other domains such as incidence of severe GVHD, and overall relapse [22]. Future studies measuring the plasma concentration of MMF and adjusting it to optimal dose might advance its superiority, and consequently, MTX might be substituted by this agent.

SOM is an impactful complication associated with MTX prophylaxis for GVHD, thus attempting to find alternative prophylaxis that is preferable to MTX in this aspect is ongoing. Sirolimus, a mammalian target of rapamycin (mTOR) inhibitor, works by inhibiting the cell cycle of T lymphocytes [62]. It has been investigated in combination with tacrolimus in several studies as GVHD prophylaxis, with inconsistent and controversial results. While two studies reported an advantage of using sirolimus instead of MTX [12, 32], others found no differences [33, 34]. Interestingly, case reports of patients receiving sirolimus as chemotherapy for solid tumors showed that aphthous-like oral ulcerations (mTOR inhibitor-associated stomatitis “mIAS”) may develop in these patients [64]. Currently, the role of T lymphocytes in pathogenesis of aphthous stomatitis is being recognized [65]. This raises a question whether or not the pathogenesis of sirolimus-induced mIAS that may develop after grafting is of non-specific ulceration that resembles aphthae. Further research is needed to elaborate on this point.

MD-MTX, consisting of 5 mg/m^2^/d on days 1, 3, 6, and 11, is an option suggested to decrease the MTX-related toxicity, especially oral mucositis. Our data evaluating five studies consistently supported the use of MD-MTX instead of the standard dose, as this was associated with a lower risk of SOM. Nevertheless, the efficacy of using such a low-dose scheme in prevention of acute and severe chronic GVHD may be a concern. The studies included in this review and others showed a comparable effect between mini-dose and standard-dose MTX in the prevention of acute GVHD and relapse in general, indicating that MD-MTX may replace standard dose in GVHD prophylaxis protocols [47, 49–51, 66]. A recent study investigating omitting day 11 of a mini-dose protocol unfortunately resulted in a higher risk of severe GHVD [67]. This suggests that the current protocol of MD-MTX is effective, and further optimization could be achieved by investigating combining it with various other immunosuppressive drugs. Combining MD-MTX with MMF was reported to have/showed a favorable toxicity profile compared to SD-MTX/tacrolimus [46]. Adding FA to a MD-MTX protocol may result in a further beneficial effect to avoid SD-MTX-related toxicity, especially in those who are at high risk for grade 3 or 4 mucositis (i.e., receiving myeloablative conditioning regimens), although this requires further investigation.

Pt-Cy has also shown potential as a preventative treatment for GVHD in matched allo-HCT [68]. Studies have indicated a low incidence of chronic GVHD when using Pt-Cy alone or in combination with other immunosuppressive medications [69, 70]. There have been limited research addressing oral mucosa toxicity, there have been limited studies, and we identified two studies that reported lower incidence of SOM in patients treated with Pt-Cy as GVHD prophylaxis compared to those treated with methotrexate [52, 53]. This finding aligns with the literature, which emphasizes the association between severe mucositis and chemotherapy affecting the S-phase of cell cycles, such as MTX [71]. Even though these two studies did not provide robust evidence related to Pt-Cy-related mucositis, prophylactic efficacy. Further research is needed on this regimen, focusing on the optimal dose administration and patient selection in allo-HCT. This may assist in determining the most effective intervention for prevention SOM in patients undergoing transplantation.

Palifermin is a recombinant human keratinocyte growth factor that was approved by the Food and Drug Administration (FDA) in 2004. Its purpose is to reduce the chance of developing severe oral mucositis (SOM) and decrease its duration, particularly for patients undergoing a myeloablative conditioning regimen before HCT [72, 73]. Palifermin was also investigated for the prevention of acute GVHD in allogeneic HCT recipients, but results were disappointing [74]. In our review, we found only one study on MD-MTX for the prophylaxis of GVHD that included this biological therapy in its prophylactic protocol. However, this study reported that out of 18 patients who received palifermin, 11 still developed some grade of mucositis [51]. Similarly, Jagasia et al. reported high-grade (III and IV) oral mucositis in allo-HCT patients receiving palifermin in their prophylactic GVHD regimen, indicating a lower efficacy in preventing SOM [74]. A recent systematic review conducted by the Mucositis Study Group of the Multinational Association of Supportive Care in Cancer/International Society for Oral Oncology (MASCC/ISOO) indicated a high level of evidence for using palifermin in mucositis prevention in patients with hematological cancer undergoing autologous HCT with myeloablative conditioning regimens including total body irradiation [75]. The reported controversy with respect to the efficacy of palifermin for the prevention of oral mucositis might be associated with the dosing schedule and conditioning regimens, which warrant further investigation.

Lastly, but not of less importance, is the need for attention to pretransplant high-intensity conditioning regimens that play a key role in causing an increased incidence and severity and duration of oral mucositis. Nevertheless, Chaudhry et al. conducted a systematic review and found that reduced-intensity conditioning regimens resulted in a high frequency of oral mucositis similar to that of myeloablative regimens [76]. Such inconsistency was also noted in this review, with some reports suggesting a lower rate of SOM in reduced intensity regimens [34, 37], and others did not find significant variation in incidence and duration of SOM in association with the types of pretransplant conditioning protocol [49]. This controversy is likely due to the lack of standardization of other factors, such as patient age, gender, type of stem cell transplant, and the various conditioning regimens (chemotherapy or chemoradiotherapy). Importantly, the fact that the majority of our studies included in the meta-analysis (7 out of 8) compared MTX to all comparators employed specifically myeloablative regimens, suggesting that the results observed are not affected by differences in treatment protocols or regimens, thereby confirming the validity of our findings.

It is important to consider some limitations of the present study. First, even though the data originate from randomized studies, the risk of bias of majority of RCTs was rated as having some concerns, which might lead to non-robust results. Second, we found variation in the dose and administration protocols of the prophylactic agents, which could explain the non-conclusive observations of several analyses of this review. In addition, there is scarcity in the number of studies focused on MTX-associated SOM, wherein many of our analyses only included two studies, which may lead to unreliable results.

## Conclusion

The risk of SOM associated with MTX is significantly higher than with alternative prophylactic GVHD approaches, and this can have substantial and even potentially life-threating consequences and severely impact the quality of life of affected individuals. In clinical situations where effective GVHD prophylaxis is felt to be equivalent, cyclosporine, post-transplant cyclophosphamide, and sirolimus have shown promising results with respect to reducing SOM risk when compared to MTX. This study suggested that combination of MD-MTX, MMF, and FA can be an alternative treatment for reduction or even prevention of SOM in allo-HCT recipients. This work also highlights the scarcity of RCTs with MTX intervention, underscoring the need for more well-designed studies to substantiate evidence-based recommendations in this area.

## Data Availability

All data supporting the findings of this study are available within the paper.
